# Community acceptability, participation, and adherence to mass drug administration with primaquine for *Plasmodium vivax* elimination in Southern Thailand: a mixed methods approach

**DOI:** 10.1186/s12936-023-04443-3

**Published:** 2023-01-13

**Authors:** Sayambhu Saita, Wanlapa Roobsoong, Patthanasak Khammaneechan, Phnom Sukchan, Saranath Lawpoolsri, Jetsumon Sattabongkot, Liwang Cui, Kamolnetr Okanurak, Suparat Phuanukoonnon, Daniel M. Parker

**Affiliations:** 1 Faculty of Public Health, Thammasat University, Lampang, Thailand; 2 Thammasat University Research Unit in One Health and Ecohealth, Thammasat University, Pathum Thani, Thailand; 3 Mahidol Vivax Research Unit, Faculty of Tropical Medicine, Mahidol University, Bangkok, Thailand; 4 Excellence Center for DACH, Walailak University, Nakhon Si Thammarat, Thailand; 5 Faculty of Medicine, Princess of Naradhiwas University, Narathiwat, Thailand; 6 Department of Tropical Hygiene, Faculty of Tropical Medicine, Mahidol University, Bangkok, Thailand; 7 Division of Infectious Diseases and Internal Medicine, Department of Internal Medicine, University of South Florida, Tampa, FL USA; 8 Department of Social and Environmental Medicine, Faculty of Tropical Medicine, Mahidol University, Bangkok, Thailand; 9 Department of Population Health and Disease Prevention, University of California, Irvine, USA; 10 Department of Epidemiology and Biostatistics, University of California, Irvine, USA

**Keywords:** Acceptability, Mass drug administration, Primaquine, Malaria elimination, Mixed-methods

## Abstract

**Background:**

Mass drug administration (MDA) with primaquine (PQ) is being considered for accelerating *Plasmodium vivax* elimination in remaining active foci. This study aimed to determine the acceptability of MDA with PQ in malaria endemic villages in a malarious setting in the South of Thailand undergoing MDA with PQ.

**Methods:**

A cross-sectional mixed-methods approach was conducted in seven malaria endemic villages where MDA with PQ was implemented. The data were collected from community villagers and health workers using structured questionnaires, in-depth interviews, and focus group discussions. Descriptive statistics and logistic regression models were used for quantitative data analysis. Thematic analysis was applied for qualitative data.

**Results:**

Among a total of 469 participants from the MDA villages, 293 participants were eligible for MDA with PQ and 79.86% (234) completed 14-days of PQ. The logistic regressions indicated that males (adjusted odds ratio: 2.52 [95% confidence interval: 1.33–4.81]) and those who are farmers (2.57 [1.12–5.90]) were most likely to participate in the MDA. Among 293 participants in the post-MDA study, 74.06% had originally agreed to participate in the MDA with PQ while 25.94% had originally reported not wanting to participate in the MDA. Of those who originally reported being willing to participate in the MDA, 71.23% followed through with participation in the first or second round. Conversely, 93.24% of those who originally reported not being willing to participate in the MDA did in fact participate in the MDA. Factors contributing to higher odds of agreeing to participate and following through with participation included being male (1.98 [1.06–3.69]) and correctly responding that malaria is preventable (2.32 [1.01–5.35]) with some differences by village. Five key themes emerged from the qualitative analyses: concern about side effects from taking PQ; disbelief that malaria could be eliminated in this setting; low overall concern about malaria infections; misunderstandings about malaria; and a general need to tailor public health efforts for this unique context.

**Conclusion:**

While the reported likelihood of participating in MDA was high in this setting, actual follow-through was relatively moderate, partially because of eligibility (roughly 71% of those in the follow-up survey who originally agreed to participate actually followed through with participation). One of the largest concerns among study participants was PQ-related side effects—and these concerns likely heavily influenced participant adherence to the MDA. The results of this study can be used to tailor future MDAs, or other public health interventions, in this and potentially other similar settings.

## Background

All nations of the Greater Mekong Subregion (GMS) have committed to eliminating malaria by the year 2030 [1]. Eliminating malaria from the region will require a suite of public health interventions, including: ensuring a strong early diagnosis and treatment system is in place [2, 3]; strong surveillance [4, 5]; and targeting asymptomatic infections which can lead to onward transmission of malaria [6] and which are unlikely to be detected by normal diagnosis and treatment systems [7].

Most malaria elimination efforts have focused on *Plasmodium falciparum* malaria. However, *Plasmodium vivax* represents a major epidemiological burden in the region and in order to truly eliminate malaria it will be necessary to address this species as well [8]. A major challenge to eliminating *P. vivax* are dormant hypnozoites, which may cause 50% to 80% of all reported *P. vivax* cases [9]. In order to address vivax hypnozoites it is necessary to administer an 8-aminoquinoline such as primaquine (PQ). Individuals with glucose-6 phosphate dehydrogenase (G6PD) deficiencies can experience hemolysis if they are administered PQ, and many populations in malarious regions have high prevalence of G6PD deficiency [10–12].

Malaria remains an important cause of morbidity in Thailand and malaria transmission in the nation is concentrated in forested areas along the international borders, especially in the western, northeast, and southern provinces [13]. As the burden of falciparum malaria has decreased, the contribution to the overall malaria burden from *P. vivax* has increased [14]. In order to eliminate malaria, it will be crucial to focus on vivax malaria as well.

One approach that is being considered in some GMS nations is targeted mass drug administration (MDA) with PQ to clear the hypnozoite reservoir and disrupt transmission. MDA with PQ has been used in several nations (including Azerbaijan, Tajikistan, Afghanistan, China, the People’s Democratic Republic of Korea, Turkmenistan), mostly targeting temperate strains of *P. vivax* [15–17]. In many cases MDA with PQ was successful at reducing *P. vivax* transmission [16–18] and where adequate healthcare infrastructure, vector control, and community participation were in place, led to long-term reductions [16, 19–22]. Relatively few severe adverse events were reported, despite some of these programs being implemented in regions with high prevalence of G6PD deficiency. MDA with PQ is, therefore, being considered for accelerating malaria elimination in the remaining active foci of *P. vivax* transmission in the GMS [23, 24].

Several factors contribute to the effectiveness of MDA, including participation by the targeted population (which impacts overall coverage of the anti-malarials). This paper presents the results of a mixed methods study among community members and public health workers in villages that were undergoing MDA with PQ in the malaria endemic South of Thailand. This study was done before and after MDA, and presents results on community members reported acceptability of MDA (i.e. will you participate in the MDA?) and subsequently whether or not they did participate in MDA. With interest from the Ministry of Public Health of Thailand in the implementation of MDA with PQ, this project provides comprehensive information regarding the acceptability and perceived feasibility of implementing MDA with PQ. The results are useful for understanding potential barriers to MDA with PQ, as well as for helping to plan the potential scale-up of MPPT in Thailand to reach the goal of elimination.

## Methods

### Study design and location

**Fig. 1:**
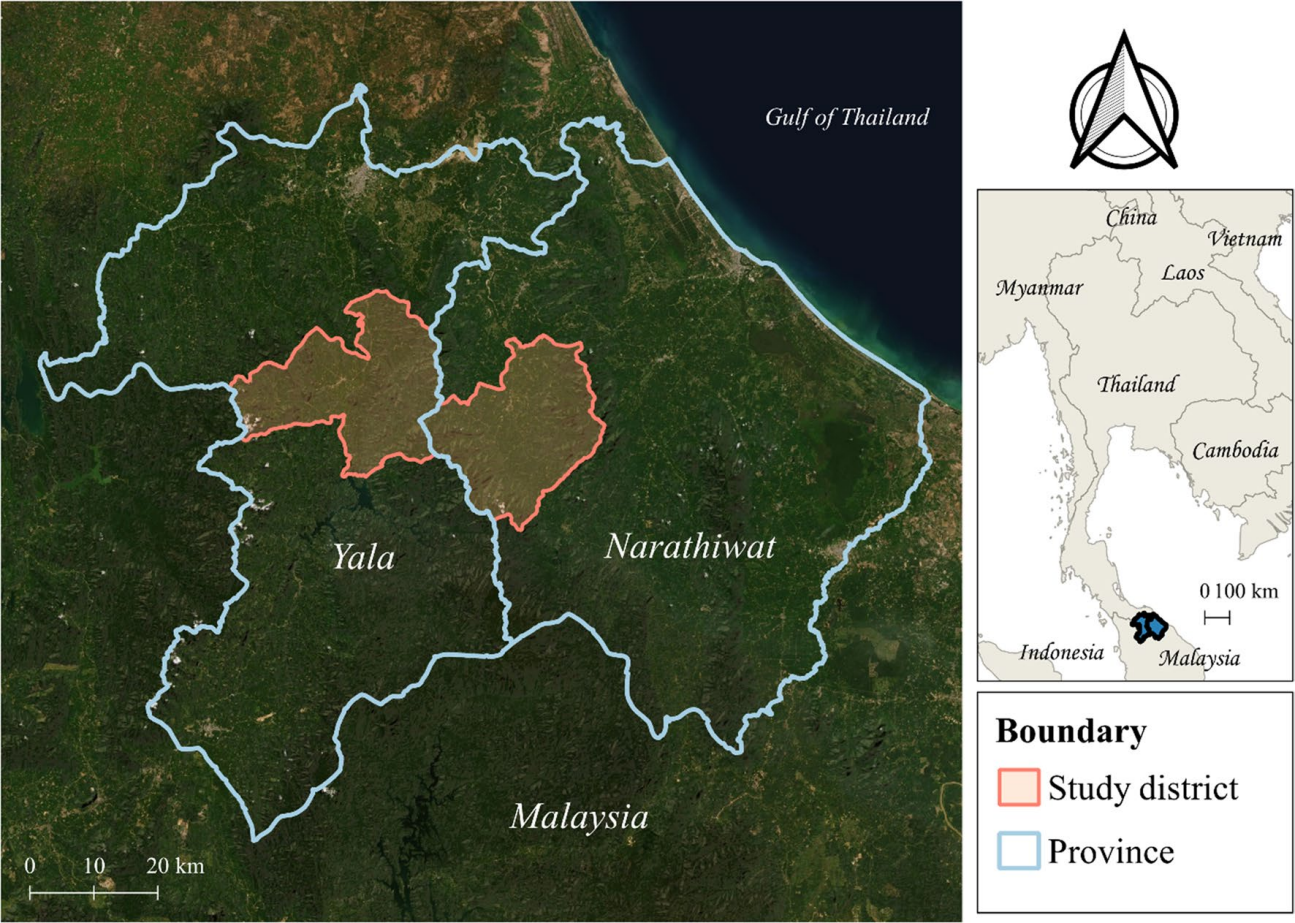
Study sites in Yala and Narathiwat Provinces of Southern Thailand

This study used a cross-sectional mixed-methods approach (including both quantitative and qualitative components). The study was conducted in 7 malaria endemic villages; 2 villages were selected in Narathiwat Province and 5 villages were selected from Yala Province (Fig. 1), both provinces in malaria endemic Southern Thailand which borders Malaysia. In 2019, total malaria cases in Yala and Narathiwat provinces were 1,368 and 70 cases, respectively. *P. vivax* in this region is widespread and there are concerns about chloroquine and antifolate resistant *P. vivax* malaria and artemisinin resistant *P. falciparum* malaria [25].

The MDA with PQ was implemented in all of 7 study villages using a step-wedge approach. The MDA was done using a low dose of primaquine (0.25 mg/kg) over 14 days, with direct observation. All individuals were tested for G6PD deficiency. G6PD deficient individuals; children < 7 or anyone weight < 15 kg; anemic individuals (with hemoglobin level < 8 g/dL); pregnant or lactating women; and anyone who self-reported previous adverse symptoms from taking primaquine were excluded from the MDA. The first round of MDA with primaquine was done in September 2019 and a second round was done in August 2020. Detailed results from the MDA will be reported elsewhere.

### Sample selection

For the pre-MDA component of the study, household leaders or representatives were recruited from 7 study villages [5 village in Yala and another 2 from Narathiwat (Fig. 1)]. Inclusion criteria included being at least 18 years of age, living in the respective village for at least a year, and being willing to answer questions during an interview. The sample sizes of participants were calculated based on an assumed 50% acceptance (agreement to participate in MDA) with 5% acceptable error and 95% confidence level, and an estimated 30% non-response. The participants in the quantitative study were selected using sample random sampling on a name list of the villagers. In total, a sample size of 500 participants were attempted and final sample of this study included 469 participants.

**Fig. 2:**
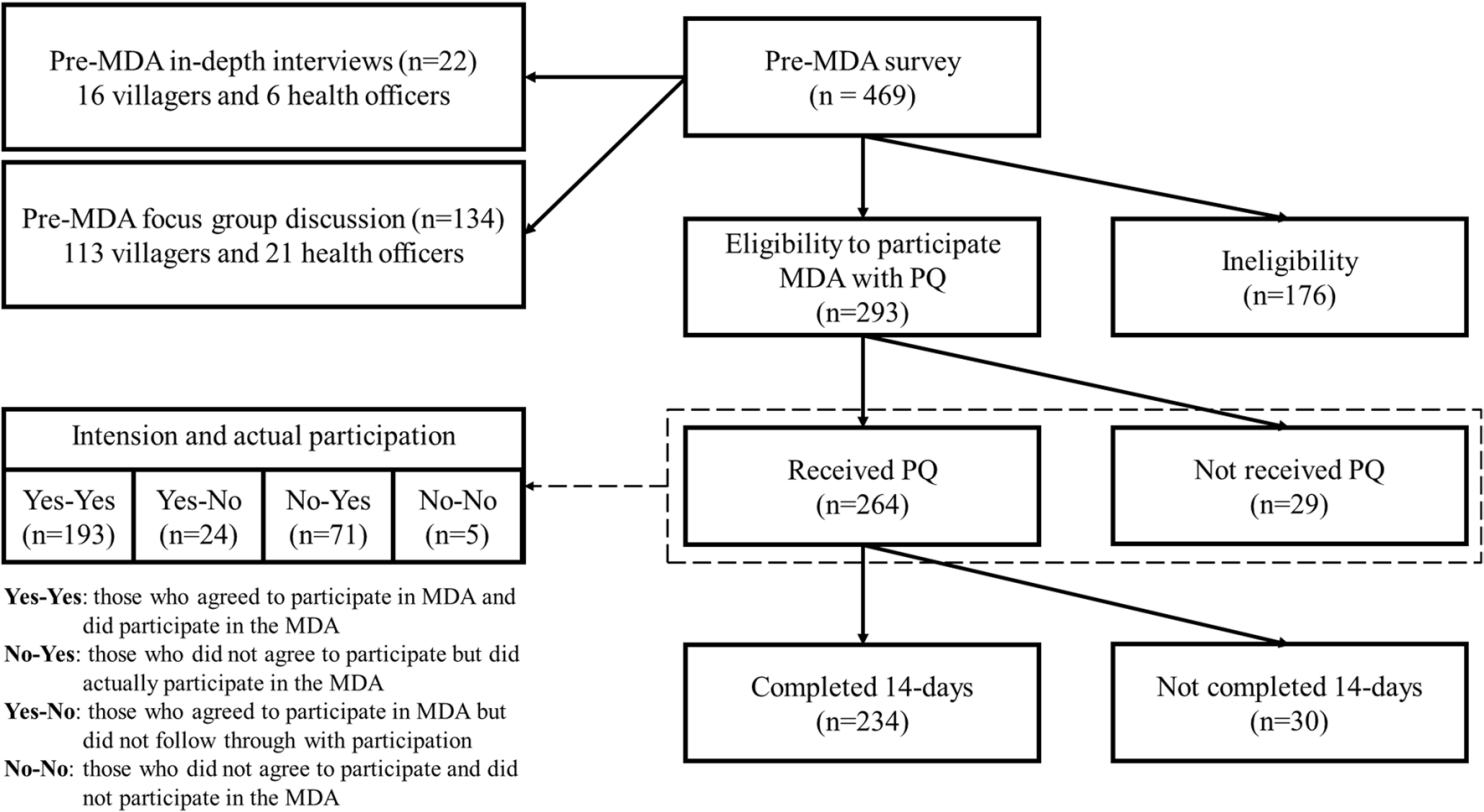
Study diagram, including the pre-MDA quantitative survey; pre-MDA qualitative interviews and focus group discussions (FGDs); and post-MDA quantitative survey

For the post-MDA component, study participants (one representative per household) from the same households in the pre-MDA study were re-contacted for follow-up surveys. A total of 293 participants were included for the post-MDA component study [covering 62.47% of pre-MDA participants (Fig. 2)].

### Quantitative data

Structured questionnaires were developed and deployed in each of the study villages. The questionnaire included 4 main parts. The first consisted of general demographic characteristics, including: gender, age, religion, education level, occupation, and experience of malaria infection. The second part consisted of questions about general malaria knowledge, including: malaria causes, diagnosis, symptoms, treatment, prevention, and reinfection. Overall knowledge was summarized by calculating a score (highest score being 7 and lowest being 0). The third part investigated perceptions about malaria, developed following the health belief model, and including questions about perceived susceptibility (5 items), perceived severity (4 items), perceived benefits of MPPT (2 items), and perceived barriers to MPPT (3 items). Again, a score was calculated with a maximum possible score of 42, categorized into 3 levels using Bloom’s criteria [26] with scores of 0–24 considered low perception, scores of 25–33 as moderate perception, and scores of 34–42 as high perception. The fourth component of the study addressed general acceptability of MDA with PQ, including whether or not interviewed participants or their household members agreed to participate in the MDA with PQ. Data from the actual MDA administration were then used to assess concordance and discordance between responses in the pre-MDA survey and actual adherence to and participation in the MDA.

### Qualitative data

Community members and health officials working in the selected villages were targeted for the qualitative study for the pre-MDA component of this project. The data were obtained by in-depth interviews (IDI) from 16 villagers and 6 health officers and by focus group discussions (FGD) with 113 villagers and 21 health officers. Inclusion criteria for health care workers included having work experience of at least one year of service. The villagers in the FGD were purposely recruited for this study (Fig. 2). Inclusion criteria among community members included being above 18 years old, living in the respective village for at least five years.

### Data management and analysis

The quantitative data were entered into Microsoft Excel 18 and analysed using the IBM Statistical Package for the Social Sciences for Windows, version 23 (IBM Corp., Armonk, NY, USA). Descriptive statistics including percentage, mean, median, standard deviation, minimum and maximum values were presented. Univariable and multivariable logistic regression models were used to investigate associations between general characteristics, knowledge and attitude regarding malaria, and agreement to participate in the MDA. For the post-MDA analysis, logistic regressions were used to assess predictors of participation in the MDA, and to assess discordance between reported intention to participate (or not to participate) and actual participation (either did or did not participate). Three main categories were defined for this part of the study: those who agreed to participate in MDA and did participate in the MDA (categorized as “Yes–Yes”); those who did not agree to participate but did actually participate in the MDA (categorized as “No–Yes”); those who agreed to participate in MDA but did not follow through with participation (categorized as “Yes–No”). The hypothesis was that socio-demographic factors; as well as experience, knowledge, and perceptions about malaria; would likely contribute to both a participant’s reported willingness to participate in the MDA and for concordance between their reported acceptability and actual participation. All variables were therefore included in the multivariable regressions. Crude odds ratios (OR) and adjusted odds ratios (AOR) were calculated with 95% confidence intervals (CI).

The qualitative interviews were conducted in the Thai language and were translated into English by SS. The correctness and completeness of transcripts were thoroughly checked by SP and DMP. The intended meanings of the original texts have also been ensured by comparing them with English translations. After that, each transcript was coded line-by-line and coded by key topics or themes. Thematic analysis was used to analyse the interviews, with key themes emerging through reading and re-reading the texts [27]. The constructed texts were finalized and confirmed among all authors until all the conclusions were mutually agreed upon.

### Ethics review

Ethical approval for this study was received from the Ethics Committee of the Faculty of Tropical Medicine, Mahidol University, Bangkok, Thailand. Number of Certificate of Ethic Approval is MUTM 2019-044-01.

## Results

A total of 469 community members were included in the pre-MDA component and a total of 293 were included for the post-MDA component of the study (Fig. 2). The qualitative interviews provide insight into some of these quantitative findings.

### General participant characteristics

For the quantitative pre-MDA study, among the 469 participants the mean (standard deviation) age was 44.30 ± 15.10 years. Most participants were female (53.52%), of the Islamic faith (86.78%), roughly half had primary education only (47.55%), and the majority were married (80.81%). The most common occupation among participants was agriculture (48.19%) and most had experienced malaria infection (71.43%). Among the survey participants, there were 293 participants who were eligible for MDA (and 176 who were ineligible). Out of the 293 participants who were eligible, 29 participants did not take any PQ (Table 1).

**Table 1:** General characteristics of study participants (n = 469)

Characteristics	n	%
Gender		
Female	251	53.52
Male	218	46.48
Age (years)		
18–30	96	20.47
31–40	109	23.24
41–50	100	21.32
51–60	99	21.11
> 60	65	13.86
$\overline{\mathrm{x }}\pm \mathrm{SD }$	44.30	± 15.10
Religion		
Buddhism	62	13.22
Islamism	407	86.78
Education level		
Illiterate	49	10.45
Monastery education	30	6.40
Primary school	223	47.55
Secondary school	156	33.26
University and above	11	2.35
Occupation		
Unemployed	9	1.92
Farmers	226	48.19
Merchants	26	5.54
Government officer	14	2.99
Housewife	63	13.43
Student	15	3.20
General labors	116	24.73
Marital status		
Married	379	80.81
Widow/divorced/separated	40	10.66
Single	50	8.53
Experienced malaria infection		
No	134	28.57
Yes	335	71.43
Participation status		
Eligibility pass at the beginning and took PQ at least 1 dose	264	56.29
Eligibility pass at the beginning but did not take PQ for any reason	29	6.18
Ineligibility in MDA	176	37.53

In the qualitative interviews, a total of 73 villagers and health staff from Yala Province participated and a total of 83 villagers and health staff from Narathiwat Province participated. Among the participants from Yala Province, 56% were female (41 out of 73) and the median age was 33 (min = 18 and max = 68). Among participants from Narathiwat Province the median age was 38 (min = 18 and max = 75) and 54% were female (45 out of 83).

### Knowledge of and perceptions about malaria

The majority of participants could accurately explain how people acquire malaria infections (58.00%), describe malaria symptoms (74.20%), and reinfection (75.05%). Most of the participants agreed that malaria infections can be prevented (81.66%) and treated (88.27%). Only 37.53% were able to accurately describe malaria diagnosis (Table 2). Moreover, participants were likely to report that anti-malarials could result in side effects and 40% mentioned concerns with taking anti-malarials while consuming certain types of fruit (especially durian) (Table 3).

**Table 2:** Knowledge regarding malaria among study participants (n = 469)

Question topic	Correct		Incorrect	
	n	%	n	%
Malaria cause	272	58.00	197	42.00
Malaria diagnosis	176	37.53	293	62.47
Malaria symptoms	348	74.20	121	25.80
Malaria treatment	414	88.27	55	11.73
Malaria is preventable	383	81.66	86	18.34
Malaria reinfection is possible	352	75.05	117	24.95
Malaria is curable	257	54.80	212	45.20

**Table 3:** Perceptions regarding malaria among study participants (n = 469)

Perception regarding malaria	Agree		Not sure		Disagree	
	n	%	n	%	n	%
Perceive susceptibility						
Everyone can contract with malaria infection	329	70.15	74	15.78	66	14.07
Malaria causing by karma (–)	61	13.01	136	29.00	272	58.00
Malaria is a community health problem	276	58.85	145	30.92	48	10.23
Malaria prevention is duty of health officers (–)	162	34.54	146	31.13	161	34.33
Rich person usually does not infect malaria (–)	45	9.59	104	22.17	320	68.23
Perceive severity						
Children have more severe malaria than adult	94	20.04	296	63.11	79	16.84
Drinking alcohol can relieve the malaria (–)	44	9.38	177	37.74	248	52.88
Local people do not die from malaria (–)	103	21.96	256	54.58	110	23.45
Malaria can cause death	160	34.12	193	41.15	116	24.73
Perceive benefit						
House spraying can prevent malaria	249	53.09	165	35.18	55	11.73
To be radically cured, patient must completely take the treatment	302	64.39	132	28.14	35	7.46
Perceive barrier						
Anti-malarial medicines may have side effects	172	36.67	236	50.32	61	13.01
Do not take anti-malarial drugs with some fruits, such as durian or lime (–)	196	41.79	201	42.86	72	15.35
Free treatment makes people not to complete the treatment	165	35.18	189	40.30	115	24.52

(–) were the negative questions

Most study participants agreed that everyone can contract malaria infection (70.15%) and recognized malaria is a community health problem (58.85%). Participants did not agree that wealthy persons were less likely to be infected with malaria (68.23%). Furthermore, participants were not sure that children could have more severe malaria than adults (63.11%). Approximately half (54.58%) were not sure that local people do not die from malaria and that malaria can cause death (41.15%). Participants agreed that insecticide spraying can prevent malaria (53.09%) and that completely taking the medicine regimen could radically cure malaria infections (64.39%) (Table 3).

Several villagers expressed misunderstandings about malaria and malaria treatment:

“Local people believe that eating zucchini seeds can cure malaria. They think that zucchini seeds could make the blood bitter and can kill malaria parasites.” (FGD, villagers).

Public health workers also mention misperceptions about the causes of malaria:

“Some villagers didn't realize that malaria was caused by mosquito bites. They believed that drinking water will cause malaria.” (IDI, Public Health officer).

“Villagers believed that drinking water in a stream causes malaria”. (IDI, Head of Center of Vector-Borne Diseases).

### Intention to participate in MDA with PQ

Before the MDA with PQ programme began, a total of 469 participants in 7 villages were surveyed regarding whether or not they would participate in the MDA. The result showed 72.71% of participants agreed that they would take the medicine if it could lead toward eliminating malaria.

From the multivariable logistic regression, 2 variables were statistically significant predictors of agreeing to participate in the MDA, both related to knowledge about malaria (Table 4). Participants who correctly responded that malaria is preventable (AOR: 2.34 [95% CI: 1.26–4.35]) had higher odds of also agreeing to participate in the MDA. Conversely, correctly identifying malaria symptoms was negatively associated (0.39 [0.20–0.74]) with agreeing to participate (Table 4).

**Table 4:** Logistic regression for factors predicting agreement to participate in the MDA with PQ

Characteristics	Total 469	Agreed to participate (n = 341)		OR [95% CI]	AOR [95% CI]
		n	%		
Sex					
Female	251	181	72.11	Ref.	Ref.
Male	218	160	73.39	1.07 [0.71–1.60]	1.06 [0.64–1.75]
Age (years)					
> 60	65	45	69.23	Ref.	Ref.
51–60	99	72	72.73	1.19 [0.57–2.36]	1.20 [0.54–2.70]
41–50	100	78	78.00	1.58 [0.78–3.20]	1.63 [0.70–3.81]
31–40	109	77	70.64	1.07 [0.55–2.09]	1.25 [0.54–2.90]
18–30	96	69	71.88	1.14 [0.57–2.26]	1.98 [0.76–5.18]
Religion					
Buddhism	62	46	74.19	Ref.	Ref.
Islamism	407	295	72.48	0.92 [0.50–1.68]	0.46 [0.16–1.33]
Education level					
Illiterate	49	38	77.55	Ref.	Ref.
Monastery education	30	18	60.00	0.43 [0.16–1.17]	0.50 [0.16–1.62]
Primary	223	169	75.31	0.91 [0.43–1.90]	0.75 [0.31–1.82]
Secondary and above	167	116	69.46	0.66 [0.31–1.39]	0.39 [0.15–1.02]
Occupation					
Unemployed/Government Officer/Housewife/student	110	70	69.31	Ref.	Ref.
Merchant/general labor	142	108	76.06	1.41 [0.79–2.49]	1.87 [0.93–3.73]
Farmers	226	163	72.12	1.15 [0.67–1.92]	1.23 [0.63–2.40]
Marital status					
Married	379	274	72.30	Ref.	Ref.
Widow/divorced/separated	40	36	72.00	0.98 [0.51–1.90]	0.97 [0.42–2.20]
Single	50	31	77.50	1.32 [0.61–2.87]	1.22 [0.49–3.01]
Experienced malaria infection					
No	134	92	68.66	Ref.	Ref.
Yes	335	249	74.33	1.32 [0.85–2.05]	1.45 [0.83–2.53]
Knowledge					
Correct in malaria cause	272	209	76.84	1.63 [1.09–2.46]	1.55 [0.95–2.54]
Correct in malaria diagnosis	176	120	68.18	0.70 [0.46–1.06]	0.66 [0.40–1.09]
Correct in malaria symptom	348	244	70.12	0.58 [0.35–0.96]	0.39 [0.20–0.74]
Correct in malaria treatment	414	308	74.40	1.94 [1.08–3.47]	2.24 [0.98–5.11]
Correct in malaria preventable	383	294	76.76	2.74 [1.69–4.46]	2.34 [1.26–4.35]
Correct in malaria reinfection	352	266	75.57	1.73 [1.11–2.71]	1.04 [0.58–1.87]
Correct in malaria curable	257	198	77.04	1.62 [1.08–2.44]	1.63 [0.96–2.77]
Perception level					
Low (0–24 scores)	11	8	72.73	Ref.	Ref.
Moderate (25–33 scores)	304	217	71.38	0.94 [0.24–3.61]	0.94 [0.20–4.33]
High (34–42 scores)	154	116	75.32	1.15 [0.29–4.54]	1.10 [0.23–5.32]
Study village (province)					
BN (Yala)	100	74	74.00	Ref.	Ref.
BR (Yala)	65	45	69.23	0.79 [0.40–1.58]	0.79 [0.36–1.75]
DY (Yala)	60	46	76.67	1.15 [0.55–2.44]	0.63 [0.26–1.52]
KS (Yala)	36	19	52.78	0.39 [0.18–0.87]	0.72 [0.27–1.87]
TY (Yala)	48	32	66.67	0.70 [0.33–1.48]	0.45 [0.18–1.09]
KL (Narathiwat)	106	90	84.91	1.98 [0.99–3.96]	1.60 [0.72–3.57]
SB (Narathiwat)	54	35	64.81	0.65 [0.32–1.32]	0.39 [0.12–1.21]

The interviews and FGDs provide insight into villager willingness to participate in MDA. For example, some villagers disbelieved that malaria can be eliminated from this setting. They also expressed that malaria cases could be decreased but it might be hard to eliminate malaria because of cross-border population movements.

“Getting malaria out of the area would be difficult. Although the infection is gone, the villager can get the infection again when going into the border areas, forests, or farms.” (IDI, villager).

Several villagers described a situation in which they are used to living with the risk of malaria and whereby some see the risk of experiencing side effects from taking PQ as a larger challenge than the risk of acquiring malaria. This is a complex problem, related both to a deep understanding of what it is like to live with malaria (including symptoms and likelihood of acquiring the disease) and potentially to a need for more public health education especially incorrect understandings of how to prevent the disease. Some villagers also expressed fatalistic thinking, linked with socio-economic conditions (poverty and a need to provide income and sustenance). Some villagers felt like malaria risk is inevitable.

“Villagers have been facing the malaria problem for a long time, so they get used to it. However, they felt that it was a problem when the public health officer had the project about P.f. eradication campaign” (FGD, villagers).

“Villagers whose career is cutting durian leaves during the night, feared starvation. Even though they knew about getting malaria infection, they accepted it.” (FGD, villagers).

This quote also shows that there are strong socio-economic concerns. The villager logic here is: there is ever-present risk of malaria, if they take PQ they might feel sick and won’t be able to work, if they get unlucky and get malaria, then they can go to the malaria post and get treated. These concerns were repeated by other participants:

“The chills and other symptoms occur during the daytime and can disturb work. Then after work, the symptoms get better and returned to normal. Stopping working becomes a family burden and looks as being lazy person.” (FGD, villagers).

Another participant explained that financial concerns outweigh concerns about the risk of malaria:

“Last year, the public health authorities had a project to eliminate P. falciparum malaria. If someone has a blood test and found P. falciparum malaria, they will be given medicine to treat and receive 300 Baht. This project makes villagers want to get P. falciparum malaria to get money. While those whose blood test was found to be infected with P. vivax malaria they feel regret for not getting the money.” (IDI, villagers).

Concerns about PQ-related side effects also emerged as a key theme. The villagers expressed concerns about loss of work, which appeared more related to taking PQ than from infection with malaria parasites.

*“After taking the drug, I feel dizziness, lack of energy, and vomiting. I thought that it is a side effect of the drug.”* (FGD, villagers). Note that this is not specific to this MDA, as this FGD occurred prior to the MDA. Rather, this villager has experience with taking PQ from routine radical cure in Thailand (which includes low dose PQ for 14 days for participants who have no history of severe symptoms from PQ, have not been found to be G6PD deficient, and are not pregnant).

Villagers also mentioned concerns about antagonism between PQ and durian (a tropical fruit):

“Villagers believe that if they are eating durian, they should do not take the medicine.” (FGD, villagers).

One of the public health workers mentioned a related concern about interactions between food and PQ:

“Villagers prepare water spinach and rice to neutralize toxins if they are allergic or having side effects from the drug.” (IDI, Malaria post worker).

Both villagers and public health workers mentioned the need for increased tailoring of public health programmes for local socio-cultural contexts. For example, one villager mentioned incorporating local religious leaders into public health programmes:

“It would be even better if the Imam (religious leaders) could help educate the people when he went to pray. But it is important to educate religious leaders well so they can be confident in their transfer of knowledge about malaria.” (FGD, villages).

Again focusing on concerns about consuming both durian and PQ, one public health worker suggested conducting the MDA in a time when durian consumption is less common:

“Villagers should be given medicines in about a month before the durian season is ripe. If villagers eat durian, they will miss taking the full amount of medication.” (IDI, Malaria post worker).

One public health worker discussed a need for increased numbers of bed nets for socio-cultural and household reasons:

“The policy providing mosquito bed nets isn’t appropriate. Two people per net is not consistent with the religious context. In the Islamic context, a child older than 8 years old must sleep in a separated bed. Villagers don't use the nets because it's inappropriate size, only 70–80 cm in size, quite small and uncomfortable.” (IDI, Malaria post worker).

### Adherence to MDA with PQ

A total of 293 participants from the MDA villages were surveyed for the post-MDA analysis (Fig. 2). As a preliminary analysis of MDA participation, a logistic regression was used to look for predictors of finishing the full 14 days of PQ (‘adherence’ to MDA) among those who began the MDA (total of 264 began MDA and 234 completed the MDA). The participants were majority female (140 out of 264) and the mean age was 46.55 ± 13.81 years. Among these post-MDA study participants 88.64% (234 out of 264) completed MDA with 14-days of PQ.

The multivariable logistic regression indicated that only participants aged 51–60 [AOR: 10.43, 95%CI: 2.03–53.57] were most likely to completed MDA with 14-days PQ (Table 5). In the univariate analysis the 41 to 50 age group and those who were of the Islamic faith were likewise more likely to participate, though both of these covariates were not statistically significant in the full multivariable regression.

**Table 5:** Logistic regression for predictors of who completed the full 14-days of PQ among study participants who were eligible to participate in the MDA

Characteristics	Total 264	Completed 14-days PQ (n = 234)		OR [95% CI]	AOR [95% CI]
		n	%		
Sex					
Female	140	127	90.71	Ref.	Ref.
Male	124	107	86.29	0.64 [0.30–1.39]	0.84 [0.31–2.27]
Age (years)					
> 60	35	26	74.29	Ref.	Ref.
51–60	76	73	96.05	8.42 [2.12–33.52]	10.43 [2.03–53.57]
41–50	63	58	92.06	4.02 [1.23–13.16]	4.62 [0.92–23.31]
31–40	55	45	81.82	1.56 [0.56–4.33]	1.09 [0.27–4.45]
18–30	35	32	91.43	3.69 [0.91–15.05]	4.96 [0.61–40.24]
Religion					
Buddhism	33	25	75.76	Ref.	Ref.
Islamism	231	209	90.48	3.04 [1.23–7.55]	1.01 [0.21–4.97]
Education level					
Illiterate	29	25	86.21	Ref.	Ref.
Monastery education	17	16	94.12	2.56 [0.26–25.01]	1.81 [0.08–40.76]
Primary	136	119	87.50	1.12 [0.35–3.61]	0.78 [0.16–3.78]
Secondary and above	82	74	90.24	1.48 [0.41–5.34]	0.89 [0.15–5.13]
Occupation					
Unemployed/Government Officer/Housewife/student	42	37	88.10	Ref.	Ref.
Merchant/general labor	73	68	93.15	1.84 [0.50–6.76]	2.28 [0.45–11.65]
Farmers	149	129	86.58	0.87 [0.31–2.48]	1.42 [0.32–6.28]
Marital status					
Married	216	192	88.89	Ref.	Ref.
Widow/divorced/separated	19	16	84.21	0.67 [0.18–2.46]	0.58 [0.09–3.62]
Single	29	26	89.66	1.08 [0.31–3.85]	0.89 [0.19–4.19]
Experienced malaria infection					
No	63	54	85.71	Ref.	Ref.
Yes	201	180	89.55	1.43 [0.62–3.30]	0.98 [0.31–3.16]
Knowledge					
Correct in malaria cause	150	132	88.00	0.86 [0.40–1.87]	0.51 [0.20–1.30]
Correct in malaria diagnosis	107	93	86.92	0.75 [0.35–1.62]	1.31 [0.50–3.45]
Correct in malaria symptom	193	172	89.12	1.19 [0.52–2.74]	0.94 [0.33–2.70]
Correct in malaria treatment	240	215	89.58	2.26 [0.78–6.59]	2.85 [0.62–13.14]
Correct in malaria preventable	221	197	88.14	1.33 [0.51–3.48]	0.89 [0.24–3.33]
Correct in malaria reinfection	196	174	88.87	1.10 [0.45–2.49]	1.37 [0.39–4.82]
Correct in malaria curable	143	131	91.61	1.91 [0.88–4.14]	1.77 [0.62–5.10]
Perception level					
Low (0–24 scores)	9	9	100.00	Ref.	Ref.
Moderate (25–33 scores)	171	149	87.13	–	–
High (34–42 scores)	84	76	90.48	–	–
Study village (province)					
BN (Yala)	54	49	90.74	Ref.	Ref.
BR (Yala)	34	31	91.18	1.05 [0.24–4.73]	1.87 [0.34–10.17]
DY (Yala)	28	26	92.86	1.33 [0.24–7.32]	1.23 [0.18–8.59]
KS (Yala)	18	16	88.89	0.82 [0.14–4.62]	1.27 [0.14–11.54]
TY (Yala)	33	33	100.00	–	–
KL (Narathiwat)	65	55	84.62	0.56 [0.18–1.76]	0.80 [0.20–3.28]
SB (Narathiwat)	32	24	75.00	0.31 [0.09–1.04]	0.49 [0.07–3.26]

The adherence in full regiment of MDA with PQ still has some obstructions due to the duration of the PQ regimen and the limitation of health workers for following up and encouragement. One malaria post worker likewise mentioned that adherence to the full regimen of PQ would be dependent on direct observation:

“The villagers will take the medicine for 14 days when they know the price, the villagers did not throw it away. However, they will not continue taking it because they think they have recovered. After symptoms have ceased, they did not continue to take medicine because taking the drug for a long time causes dizziness.” (IDI, Malaria post worker).

Moreover, some villagers argued that more malaria public health workers were needed for their region to encourage people to participate and actively MDA implementation:

“There are 24 Malaria Post workers in the entire district. Campaigning for any programs is difficult (low manpower).” (FGD, villagers).

### Agreement or discordance between intention to participate and actual participation in MDA with PQ

Of the 293 participants who were eligible to participate in the MDA with PQ, 74.06% (217/293) had originally agreed to participate in the MDA with PQ while 25.94% (76/293) had originally reported not wanting to participate in the MDA (Table 6). Of those who originally reported being willing to participate in the MDA, only 71.23% (193/217) followed through with participation. Conversely, 93.24% (71/76) of those who originally reported not being willing to participate in the MDA did in fact participate in the MDA.

**Table 6:** Logistic regression for factors predicting discordance or concordance between reported participation and actual participation in MDA with PQ

Characteristics	Total 293	Yes–yes (n = 193)			Yes–no (n = 24)			No–yes (n = 71)		
		n	%	AOR [95% CI]	n	%	AOR [95% CI]	n	%	AOR [95% CI]
Sex										
Female	158	98	62.03	Ref.	16	10.13	Ref.	42	26.58	Ref.
Male	135	95	70.37	1.98 [1.06–3.69]	8	5.93	0.73 [0.23–2.31]	29	21.48	0.51 [0.26–1.02]
Age (years)										
> 60	39	23	58.97	Ref.	4	10.26	Ref.	12	30.77	Ref.
51–60	78	56	71.79	2.14 [0.77–5.99]	1	1.28	0.11 [0.01–1.27]	20	25.64	0.58 [0.20–1.70]
41–50	70	48	68.57	1.63 [0.55–4.85]	6	8.57	1.09 [0.17–6.85]	15	21.43	0.45 [0.14–1.47]
31–40	61	41	67.21	1.49 [0.51–4.38]	4	6.56	0.71 [0.11–4.42]	14	22.95	0.46 [0.14–1.52]
18–30	45	25	55.56	0.97 [0.27–3.49]	9	20.00	1.52 [0.20–11.54]	10	22.22	0.53 [0.13–2.24]
Religion										
Buddhism	37	23	62.16	Ref.	4	10.81	Ref.	10	27.03	Ref.
Islamism	256	170	66.41	0.85 [0.25–2.90]	20	7.81	–	61	23.83	1.67 [0.46–6.12]
Education level										
Illiterate	32	23	71.88	Ref.	1	3.13	Ref.	6	18.75	Ref.
Monastery education	19	9	47.37	0.39 [0.09–1.68]	2	10.53	2.00 [0.12–32.37]	8	42.11	4.73 [0.96–23.18]
Primary	150	105	70.00	0.71 [0.24–2.14]	11	7.33	1.26 [0.10–15.34]	31	20.67	2.04 [0.61–6.83]
Secondary and above	92	56	60.87	0.42 [0.13–1.39]	10	10.87	1.45 [0.11–18.49]	26	28.26	5.36 [1.39–20.59]
Occupation										
Unemployed/Government Officer/Housewife/student	52	33	63.46	Ref.	9	17.31	Ref.	9	17.31	Ref.
Merchant/general labor	81	55	67.90	0.85 [0.34–2.14]	6	7.41	0.80 [0.20–3.24]	18	22.22	1.53 [0.52–4.52]
Farmers	160	105	65.63	0.61 [0.25–1.51]	9	5.63	0.91 [0.18–4.46]	44	27.50	2.11 [0.75–5.91]
Marital status										
Married	239	157	65.69	Ref.	19	7.95	Ref.	59	24.69	Ref.
Widow/divorced/separated	24	14	58.33	0.92 [0.31–2.74]	4	16.67	1.11 [0.24–5.11]	5	20.83	0.89 [0.24–3.35]
Single	30	22	73.33	1.91 [0.68–5.34]	1	3.33	0.47 [0.04–5.29]	7	23.33	0.68 [0.23–2.01]
Experienced malaria infection										
No	73	41	56.16	Ref.	7	9.59	Ref.	22	30.14	Ref.
Yes	220	152	69.09	1.86 [0.89–3.88]	17	7.73	0.50 [0.12–2.09]	49	22.27	0.82 [0.37–1.80]
Knowledge										
Correct in malaria cause	172	117	68.02	1.17 [0.63–2.18]	9	11.05	2.99 [0.85–10.54]	33	19.19	0.47 [0.24–0.93]
Correct in malaria diagnosis	117	76	64.96	0.78 [0.42–1.46]	9	7.69	0.97 [0.31–3.05]	31	26.50	1.41 [0.71–2.80]
Correct in malaria symptom	213	138	74.79	0.53 [0.25–1.14]	16	7.51	0.89 [0.25–3.11]	55	25.82	2.05 [0.85–4.91]
Correct in malaria treatment	264	181	68.56	2.96 [0.94–9.34]	21	7.96	1.54 [0.15–16.31]	59	22.35	0.38 [0.11–1.28]
Correct in malaria preventable	244	173	70.90	2.32 [1.01–5.35]	19	7.79	0.65 [0.13–3.32]	48	19.67	0.41 [0.17–0.97]
Correct in malaria reinfection	218	150	68.81	1.14 [0.54–2.39]	18	8.26	1.28 [0.33–5.00]	46	21.10	0.80 [0.35–1.82]
Correct in malaria curable	157	111	70.70	1.96 [0.98–3.92]	12	7.64	0.75 [0.23–2.43]	32	20.38	0.48 [0.22–1.06]
Perception level										
Low (0–24 scores)	11	6	54.55	Ref.	2	18.18	Ref.	3	27.27	Ref.
Moderate (25–33 scores)	193	125	64.77	1.74 [0.40–7.58]	19	9.84	0.81 [0.09–7.37]	46	23.83	0.68 [0.13–3.59]
High (34–42 scores)	89	62	69.66	2.39 [0.51–11.25]	3	3.37	0.17 [0.01–2.19]	22	24.72	0.80 [0.14–4.56]
Study village (province)										
BN (Yala)	66	41	62.12	Ref.	10	15.15	Ref.	13	19.70	Ref.
BR (Yala)	37	27	72.97	1.99 [0.72–5.54]	3	8.11	0.47 [0.09–2.42]	7	18.92	0.73 [0.22–2.40]
DY (Yala)	32	21	65.63	0.75 [0.25–2.21]	3	9.38	0.63 [0.11–3.69]	7	21.88	1.76 [0.49–6.30]
KS (Yala)	21	8	38.10	0.78 [0.20–2.99]	2	9.52	0.60 [0.07–5.56]	10	47.62	1.60 [0.40–6.44]
TY (Yala)	35	21	60.00	0.63 [0.22–1.81]	2	5.71	0.26 [0.04–1.84]	12	34.29	3.97 [1.18–13.37]
KL (Narathiwat)	66	55	83.33	3.16 [1.15–8.67]	1	1.52	–	10	15.15	0.97 [0.33–2.87]
SB (Narathiwat)	36	20	55.56	0.69 [0.18–2.62]	3	8.33	–	12	33.33	2.65 [0.63–11.07]

This analysis included all participants who eligible to take PQ. One group (No–No; those who originally said they would not participate and also did not participate) had very small numbers (only 5) and was not included in this analysis

#### Intentioned participation and actual participation

Males were more likely to have both reported willingness to participate and to have followed through with participation in the MDA, with almost 2 times the odds when compared to females (1.98 [1.06–3.69]; Table 6). Participants who correctly responded that malaria is preventable (2.32 [1.01–5.35]) and participants in KL village of Narathiwat Province (3.16 [1.15–8.67]) were also more likely agree and follow through with MDA participation.

#### Intentioned participation but non participation

There were no statistically significant predictors of discordance among those who originally agreed to participate in MDA (n = 24, Table 6).

#### Not intentioned participation but actual participation

Participants who had secondary education and above (5.36 [1.39–20.59]) were most likely agree to participate in, but did not follow through with, MDA participation. Those who correctly responded that malaria in preventable (0.41 [0.17–0.97]) and about malaria cause (0.47 [0.24–0.93]) were less likely to have discordance in their reported participation and actual participation in MDA with PQ. The participants in TY village of Yala Province (3.97 [1.18–13.37]) were more likely agree to participate but did not follow through MDA participation.

## Discussion

The relative effectiveness of MDA is dependent on many factors, including the epidemiology of the disease (i.e. high- versus low-transmission setting), existing healthcare infrastructure (which can help with quick diagnosis and treatment if parasites are reintroduced after MDA), the anti-malarials used, and coverage of the MDA (how much of the population receives a sufficient dose) [16, 19, 28]. Community participation is therefore a crucial component of successful MDA campaigns [19, 28, 29]. Recognizing the importance of community participation, this research occurred in parallel to an MDA campaign with PQ, and sought to understand both intentions to participate in the MDA and actual participation in the MDA. This work began with asking participants whether or not they would participate in MDA, then investigating the context behind their attitudes and opinions of malaria and MDA with PQ (the qualitative component), and finally analysed discordance between villagers original stated intention to participate and their actual participation behavior.

The Health Belief Model (HBM) was developed to help understand why people do or do not adopt public health interventions and can be applied to MDA [30]. While there are variations of the HBM, the original version suggests that perceived susceptibility to a disease, perceived severity of a disease, perceived benefits of the intervention, and perceived barriers to uptake of the intervention all influence whether or not individuals will uptake or participate in a given intervention. In the present research, this can be applied to concerns about susceptibility and severity of vivax malaria, general beliefs about whether or not the intervention can achieve its stated goals (local elimination of vivax malaria), and any perceived barriers to participation in the MDA with PQ (including concerns about side effects) [21].

Following the HBM, the interviews and FGDs in this research provided several insights into participant concerns and beliefs about malaria and MDA with PQ. For example, several villagers mentioned having a history of dealing with malaria and understanding that they could be cured when they had fever and headache and went to a health post for diagnosis and treatment. Likewise, some villagers expressed doubt that malaria could be eliminated from this setting, especially as many visit places outside of their communities where they may acquire infections. This indicated that villagers are concerned about the movement of the population in the area where the infection may come from other areas, which will contribute to the ongoing malaria epidemic [31]. Finally, a common theme that emerged included worries about loss of labor because of potential side effects from taking PQ, that outweighed concerns about malaria infection [32–34].

There was some discussion about adherence to MDA with PQ in the interviews and FGDs. Two main themes emerged from these discussions. Participants noted that adherence to MDA was being influenced by potential side effects associated with taking PQ, and a potential loss of labor because of these side effects. Furthermore, there was discussion about how encouragement from health workers could increase adherence. Health care workers and local staff could engage and advocate communities, disseminate information and build the trust between the target communities and intervention [35].

Regardless of these key themes, farmers (especially males) were most likely to follow through with participating in the MDA. Conversely, women under the age of 30 were the most likely to not participate in the MDA, including originally stating that they would participate but not actually following through with participation when the MDA occurred. This finding may relate to reproductive intentions and concerns with PQ use in pregnancy or breastfeeding. Individuals with low education levels were also less likely to participate in MDA.

Another important theme that emerged from this analysis relates to tailoring public health approaches for this specific context. Concerns about the side effects of taking PQ were commonly mentioned, especially alongside consumption of fruits such as durian. This might be related to beliefs in this region about needing to balance “hot” and “cool” substances that are consumed [36]. In this situation, both PQ and durian are considered to be “hot”, and consumption of both simultaneously is thought to be dangerous [37]. People with this belief often attempt to balance “hot” and “cool” substances, for example, consuming rice and water (both considered “cool” substances) alongside PQ [36].

Furthermore, whereas Thailand is a majority Buddhist nation, this subregion is majority Muslim and socio-cultural differences can sometimes be large. Interventions and practices used in other settings may not be appropriate here. One example of this has to do with use of bed nets among children and adolescents, where children (under 8 years) should not be sharing bed nets with older individuals—meaning that using house counts for bed net distribution might not be optimal (and should consider the age and gender makeup of the household as well). Likewise, there was a strong community support network in place in these villages lead by local religious leaders (Imams). For public health efforts to succeed in this setting, it will be important to use locally relevant social structures.

There are several limitations to this study. The pre-MDA study was done among a group of participants regardless of their eligibility (partially because G6PD testing had not been completed). In particular, this study is not able to identify FGD members and their relative eligibility to participate in the MDA, or whether or not they eventually participated in the MDA. The intention of this study was to gain an understanding of the malaria situation and relative acceptability of MDA with PQ at the community level. Furthermore, low-dose PQ for 14 days is the standard of care for vivax malaria infections in this setting. It may have been interesting to investigate villager concerns about PQ as treatment rather than as part of MDA, and this study did not explore this topic. Finally, a key theme that emerged in the interviews and FGDs was PQ-related symptoms. This study also did not investigate specific symptoms or their causes (i.e. haemolysis), aside from attempting to understand general villager concerns, how they related to agreeing to participate in MDA, and in the local malaria context.

## Conclusions

While reported likelihood of participating in MDA was high in this malaria endemic setting, actual follow-through was relatively moderate (roughly 71% of those in the follow-up survey who originally agreed to participate actually followed through with participation). Through qualitative interviews and FGDs, community members mentioned relatively low concern about acquiring malaria infections in comparison to concerns about PQ-related side effects. Some likewise mentioned doubts about actually being able to eliminate malaria from this setting. Furthermore, both villagers and public health workers discussed the importance of taking local socio-cultural norms into consideration when implementing public health interventions in this setting. The results of this study can be used to tailor future MDAs, or other public health interventions, in this setting and may help with strategies and overcoming challenges in other settings where targeted efforts will be used to eliminate *P. vivax*.

## Data Availability

All data generated or analysed during this study are included in this published article.

## Abbreviations

PQ: Primaquine
G6PD: Glucose-6-phosphate dehydrogenase
*P. vivax*: *Plasmodium vivax*
*P. falciparum*: *Plasmodium falciparum*
GMS: Greater Mekong Subregion
MDA: Mass drug administration
MPPT: Mass primaquine preventive treatment
IDI: In-dept interview
FGD: Focus group discussion
OR: Odds ratios
AOR: Adjusted odds ratios
CI: Confidence interval
